# A general factor for trust?: Testing latent factor structures of trust across institutional and interpersonal contexts

**DOI:** 10.1371/journal.pone.0335172

**Published:** 2025-10-24

**Authors:** Vincent O. Mancini, Darren M. Moroney, Jill A. Howieson

**Affiliations:** 1 UWA Law School, University of Western Australia, Perth, Western Australia; 2 The Kids Research Institute Australia, Perth, Western Australia; University of Valencia: Universitat de Valencia, SPAIN

## Abstract

**Background:**

The literature is replete with multi-dimensional self-report assessments of trust. It is not clear whether these dimensions are statistically distinguishable across institutional and interpersonal contexts, respectively.

**Aim/s:**

We sought to provide empirical insights that might permit researchers to refine the conceptualisation and dimensionality of trust, as well as provide suggestions for institutions or individuals hoping to cultivate trust. Specifically, we aimed to test whether evidence for a *general* trust factor would emerge in relation to trust in institutions and other people.

**Methods:**

588 adults completed an online survey assessing dimensions of trust measured in institutional and interpersonal contexts.

**Results:**

Confirmatory factor analyses (CFAs) revealed that a ‘many-factor’ correlated model provided the best fit statistics in both interpersonal and institutional contexts. Higher-order and bi-factor models also produced excellent fit. Exploratory structural equation modelling (ESEM) revealed a high-degree of item cross-loadings, suggesting that the tested trust dimensions were not as distinct as predicted. The bi-factor ESEM model found that all items had significant loadings on a general factor, supporting the notion of a general trust factor. This effect appeared more persuasive in interpersonal contexts, relative to institutional contexts.

**Implications:**

Trust-related dimensions may not merely be distinct, correlated constructs. Statistical evidence produced in the current study aligns with the suggestion that people’s trust-related perceptions may, in part, be influenced by a general factor. We propose the theory of epistemic trust as a candidate for interpreting the general trust factor.

## Introduction

Conceptualising and operationalising *trust* continue to be perplexing endeavours for researchers, policymakers, and the community. In many ways, *trust* is ineffable, yet it remains fundamental to our ability to interact with the surrounding world and its inhabitants. It is therefore unsurprising that a breadth of disciplines (and their sub-disciplines), including the sciences of human behaviour, sociology, psychology, psychiatry, health care, economics, artificial intelligence, international relationships, politics, and policy [1–4] remain interested in understanding how trust applies to their area of enquiry. In this study, we sought to provide empirical insights that might permit researchers to refine the conceptualisation and dimensionality of trust, as well as provide suggestions for institutions or individuals hoping to cultivate trust. Specifically, we aimed to test whether evidence for a *general* trust factor would emerge in relation to trust in institutions and other people, respectively.

The multidisciplinary relevance of trust has resulted in each discipline developing different ways of defining trust, using different terminology to describe it, and taking different approaches to its investigation [5,6]. Thus, anyone interested in arriving at a comprehensive, contemporary understanding of trust need first navigate what Metlay [7] has described as a *‘conceptual quagmire*’. As an example, one study by McEvily and Tortoriello [3] identified over 120 measures of trust in institutions (e.g., governments and organisations), referred to as *organisational* (*institutional) trust* [8]. Few of these instruments had been thoroughly validated, and the authors reported considerable conceptual overlap in the specific ‘dimensions’ of trust that were assessed. Specifically, 38 conceptually distinct dimensions were identified across these measures, and each of the instruments appeared to assess a different subset of these (see p.34).

No known study, similar in style to the study by McEvily and Tortoriello [3], has comprehensively reviewed studies using self-report instruments for the measurement of *interpersonal trust*, the second broadly accepted category of trust, defined simply as trust in individual people [8]. The interpersonal trust literature is also replete with various instruments, grounded in different methodologies, capturing different dimensions of trust [9–11], thus it is likely that a similarly opaque pattern would be revealed. The work by McEvily and Tortoriello [3] does, however, point to one potential approach to seeking an understanding of trust, and that is to seek clarity regarding the (discipline-agnostic) dimensions of trust. Inspired by this challenge, our study aimed to adopt such an approach.

### Empirical investigations of trust

In 2017, the Organisation for Economic Co-operation and Development (OECD) produced their *Guidelines on Measuring Trust* [8] with the intention of aiding the development of improved measurements. Among its recommendations, the Guidelines identified what was already relatively well known, namely that, despite the variety of definitions and measures of trust that had been developed to suit specific contexts, the self-report survey-based approach seemed to have remained a relative staple in the field. This was largely attributable to the efficiency and scale at which surveys can be distributed, calculated, and interpreted [8], and informed the data collection methodology employed in the current study.

Prior to the landmark OECD report, and after its publication, a relatively large body of literature developed through survey research and a range of advanced quantitative methods to examine conceptual distinctions between purported dimensions of trust via their statistical relationships. Studies have also sought to integrate theories of trust, employing statistical analyses to determine if the refined conceptualisations are empirically supported [12,13]. Below, we report on some key insights from three commonly used quantitative techniques to assess the dimensionality of scale-based trust instruments, namely *exploratory factor analysis* (EFA), *confirmatory factor analysis* (CFA), and *exploratory structural equation modelling* (ESEM).

**Fig 1:**
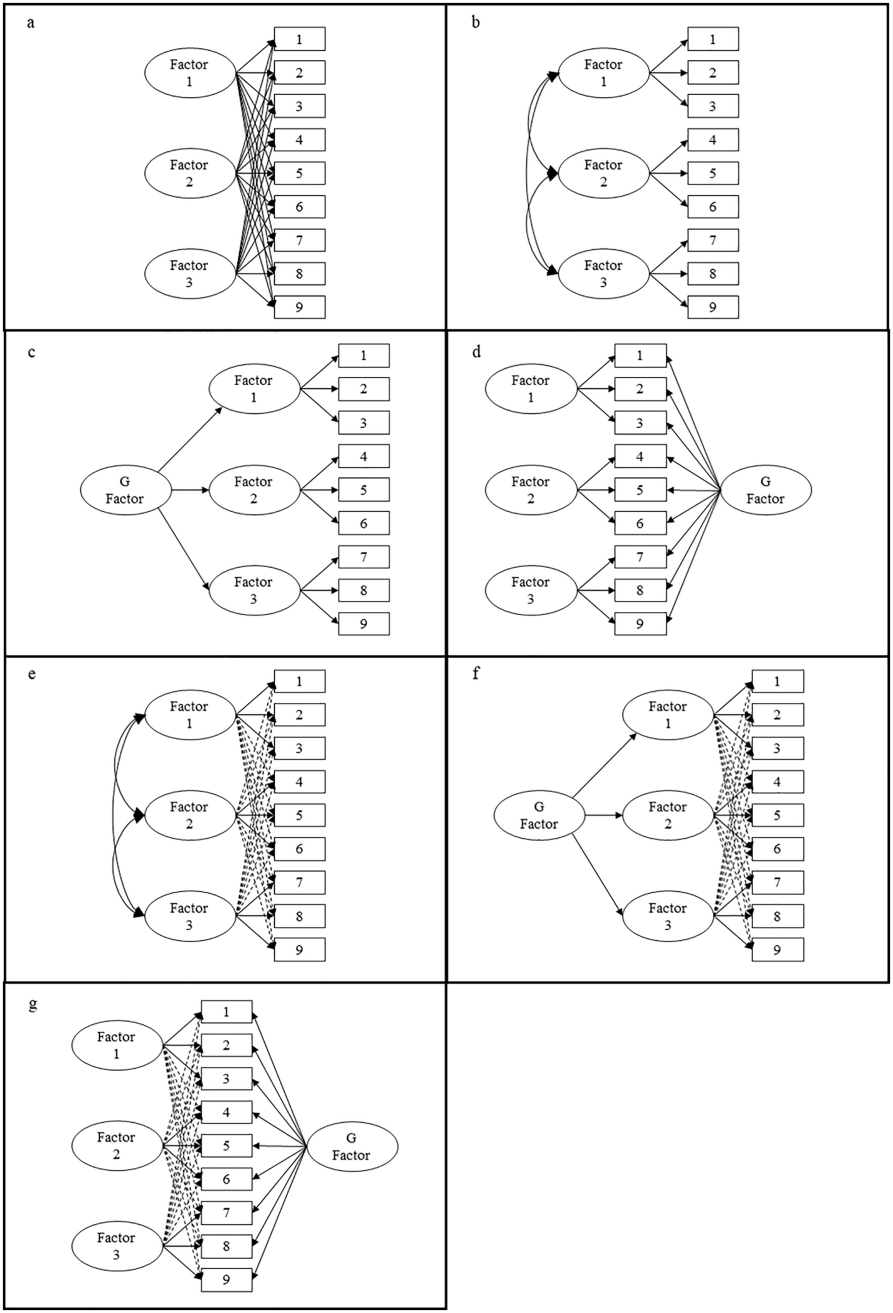
Example visual representation of exploratory factor analysis (1a), confirmatory factor analysis (1b), higher-order confirmatory factor analysis (1c), bifactor model (1d), exploratory structural equation model (ESEM: 1e), higher-order ESEM (1f), and bifactor ESEM (1g).

**EFA.** The EFA method is typically reserved for studies whose focus is on piloting a new instrument, reducing the number of items in an instrument, or determining which latent factor an item is an indicator of (see Fig 1a). EFA uses the correlations between survey *items* (often statements, or questions posed to a participant) to engage in a data-driven approach to identifying (a) the number of potential factors (i.e., dimensions) that should be retained in an explanatory model, and (b) which items load onto each retained factor. Researchers then name these factors (dimensions) based on the thematic similarities between statistically clustered items [14].

An illustrative example of EFA is Gregory, Nyein [15], who reported the results of an EFA performed on an 11-item measure of ‘patient trust in healthcare professionals’, which they had modified for use in a hospital setting. The EFA revealed a two-factor solution, with items loading on to one of two latent factors (dimensions), which were labelled *Cognitive Trust* and *Affective Trust*, respectively. Another example is the recently published *Epistemic Trust, Mistrust and Credulity Questionnaire* (ETMCQ) by Campbell, Tanzer [16]. The results of this EFA revealed that each item had distinct loadings on one of three latent factors (dimensions), which were labelled *Epistemic Trust, Mistrust,* and *Credulity*, respectively.

**CFA.** In contrast to the data-driven approach of the EFA method, the CFA method relies on a theory-driven approach to explore proposed relationships between latent factors (dimensions). Researchers generate a theory-driven explanatory model that specifies the number of dimensions and pattern of item loadings *a priori*. The model is then empirically tested to see whether it provides a good ‘fit’ for the data, or whether an alternative factor structure is required. For example, Campbell, Tanzer [16] and Gregory, Nyein [15] performed CFA in their studies, once they had developed the initial factor structures using EFA (see Fig 1b). Of particular relevance to any investigation of trust, where there is a lack of clarity about what its specific dimensions might be, and how they might be related [6], CFA also provides the opportunity to compare different factor structures and their ability to account for the data.

Previously, PytlikZillig, Hamm [17] integrated trust-related survey data from four separate institutional domains (police, local governance, natural resources, and state government), with sample sizes ranging from 399 to 1033 participants, and enlisted CFA methods to compare seven alternative factor structures (see p.114). The least complex factor structure was a two-factor, *first-order* model, whereby first-order factors are those indicated by observed items. The most complex model comprised 12 conceptually distinguishable factors (dimensions) of institutional trust and was referred to as the *many-factor* first-order model. The dimensions had been identified in a previous review of the literature, and were labelled *dispositional trust*, *bias*, *care*, *competence*, *cynical beliefs*, *fairness*, *honesty*, *legitimacy*, *loyal trust*, *shared values*, *unspecified trust*, and *voice* [18]. The study revealed that, across institutional domains, the *many-factor* model produced the best fit to the data. Similarly, Hamm and Hoffman [19] collected data from an instrument assessing institutional trust. CFAs in their study showed that of the tested first-order models, the *many-factor* model, which comprised six dimensions labelled *dispositional trust, care, competence, confidence, procedural fairness,* and *values similarity*, provided the best fit to the data, when compared to the less complex two-factor model. Despite their compositional differences, these findings appear to point to a *many-factor* model as the most appropriate way to conceptualise the underlying dimensions of trust. However, PytlikZillig, Hamm [17] and Hamm and Hoffman [19] also applied a *higher-order CFA* approach to their studies, which offered another perspective.

A *higher-order* (or ‘hierarchical’) CFA approach is relatively common in mental health [20] and intelligence research [21], whereby higher-order factors are indicated by first-order factors, rather than observed items directly (see Fig 1c). Hamm and Hoffman [19] described three ways in which higher-order models potentially offer advantages over first-order models. First, higher-order models can help to resolve overlap issues with strongly correlated latent constructs, which are relatively common in trust research. Second, they can allow first-order factors to remain distinct and recognisable whilst explicitly representing their covariation as a single factor. Third, the identification of a higher-order factor can be used to predict variance in an outcome not otherwise explained by the first-order factors.

The PytlikZillig, Hamm [17] and Hamm and Hoffman [19] studies evaluated higher-order models across a total of five data analyses. Apart from one higher-order model that did not converge, likely due to sample size constraints, the analyses were consistent in that the higher-order model produced good fit statistics according to conventional cut-off scores [17,19]. While the higher-order model fit statistics were not superior to the first-order many-factor models, Hamm and Hoffman [19] argued that a higher-order model could still be considered the better representation of the data on the grounds that a) the poorer fit of the higher-order model was expected because fit statistics often penalise more complex models, b) the overall reduction in model fit in the higher-order model was small, though still above conventionally acceptable cut-offs, and c) the higher-order factor could predict other important outcomes that were not predicted by a many-factor model. This early work was integral to the current paper.

In addition to higher-order factor models, *bi-factor* models present a relatively new approach to the exploration of structural models. Unlike the hierarchical structure of a higher-order model, where a higher-order factor is indicated by first-order factors that are, in turn, indicated by observed items, a bi-factor model instead argues that all items (i.e., observed variables) are indicated by both a *general* factor and first-order factors. Essentially, items can indicate the general factor directly, rather than via the first-order factors (see Figs 1d-g). Bi-factor models have been shown to provide better fit to data than higher-order models [22] and easier interpretation of the relationships between specific factors with other variables [23]. They can also produce general and specific factor scores that can be used to predict outcomes of interest [24].

**ESEM.** The exploratory structural equation modelling (ESEM) approach has emerged recently as a compelling alternative to understanding relationships between first-order factors. ESEM approaches can be used to analyse many-factor, higher-order, and bi-factor models. Critically, ESEM allows researchers to specify a model that has items loading on to their intended first-order factors (i.e., CFA), but also allows items to load on to other first-order factors, somewhat harnessing the advantages of both confirmatory and exploratory approaches. These properties of ESEM can help to overcome issues associated with CFA approaches where the first-order factors are not very well-established, or where there is contention concerning the properties of specific latent constructs, as is the case in the trust literature.

Following Tomlinson, Schnackenberg [12], who looked to bridge two predominant conceptualisations of a related concept, namely *trustworthiness*, Lee, Alarcon [13] provided some evidence for the potential utility of a bi-factor model in a trust context. Lee, Alarcon [13] combined the items in the Mayer, Davis [25] three-factor model with the items in the Mayer, Davis [25] two-factor model and employed ESEM to test potential explanatory models. Lee, Alarcon [13] found that *ability*, *benevolence*, and *integrity* emerged as specific dimensions, but as part of a bi-factor model where all items were also indicated by a general ‘*trustworthiness*’ factor. This suggested that participants, in part, employed a *general factor* when responding to items on the trustworthiness scales used in their research. We were interested in exploring whether a similar finding would hold for trust.

### A general factor and epistemic trust

The real-world value of any statistical model of trust lies in the interpretation of the model in terms of the potential *theoretical* mechanisms that have generated it and may elucidate its implications. Here, we propose that *epistemic trust* theory might help explain any relationships between dimensions of trust and lend support to hypotheses about a *general factor*. Initially discussed by Fonagy, Luyten [26], epistemic trust has been defined as “*an evolutionarily prewired capacity to identify knowledge that is conveyed by others as significant, personally relevant, and generalizable to other contexts*” [27]. The capacity to trust epistemically starts forming early in development within attachment relationships, as a neurological *‘superhighway’* that facilitates the ability to appraise whether social information conveyed by others can be trusted (or not) evolves in the mind. Contemporary understandings of epistemic trust position this capacity as a pre-requisite to forming trust across contexts [27]. As such, we believe that epistemic trust theory might help guide the interpretation of quantitative results and complement previous studies that have promoted the statistical advantages of higher-order and bi-factor models [17,19].

### The present study

Our study built upon the dimensions previously operationalised in the study by PytlikZillig, Hamm [17] and employed methodologies comparable to those employed by these authors and Hamm and Hoffman [19]. We pursued *three* areas of investigation with the aim of providing insights that might permit researchers to refine the conceptualisation and dimensionality of trust, as well as provide suggestions for institutions or individuals hoping to cultivate trust.

The first area of investigation pertained to advancing knowledge by applying the rigorous CFA methods employed in institutional trust research to interpersonal trust, and then expanding both via ESEM methods, to identify any similarities or differences between the institutional and interpersonal contexts. The second area of investigation pertained to advancing knowledge by considering higher-order factors, which has seldom been undertaken in the trust literature. We aimed to achieve this by comparing the fit of different first-order, higher-order, and bi-factor models of trust. Two of the most comprehensive assessments of the factor structure of trust measures [17,19] used CFA approaches that fixed the loadings of observed variables on non-intended first-order factors to zero. Thus, it was not possible to discern whether these observed items might have clustered together in ways different to those that the researchers originally considered. The third area of investigation pertained to advancing knowledge by incorporating theoretical advances within the trust literature, namely the theory of *epistemic trust*, to propose a rationale for investigating whether a *general* trust factor might explain the relationships between either the latent *trust-specific* dimensions (in higher-order ESEM) or observed items (in bi-factor ESEM). To test the above aims, we developed the following hypotheses:

- H1: A ‘many-factor’ first-order model of institutional or interpersonal trust will provide better fit to the data compared to more parsimonious first-order models.
- H2: Higher-order and bi-factor CFA and ESEM models of institutional or interpersonal trust will provide adequate fit to the data based on conventional model fit statistics.
- H3: In higher-order and bi-factor ESEM models of institutional or interpersonal trust, each item will have a significant loading on its predefined *specific* trust factor and a *general factor*, and non-significant loadings on the remaining *specific* trust factors.

## Method

### Participants

The study sample comprised 588 adults (293 females; 294 males; 1 undisclosed gender) aged 18–84 years (*M* = 33.90 years, *SD* = 11.67 years). All participants were residents of Australia (80.8%) and New Zealand (19.2%) at the time of the research, with 77.6% of the sample also born in these countries. The sample were all proficient in the English-language. Most of the sample were employed at either a full-time (58.5%) or part-time (30.6%) capacity. The remaining participants were either not in paid work (e.g., retired, unable to work, seeking employment, or due to start a job in the next month) or studying. Most participants (75% had at least completed their secondary education).

Participants were recruited via convenience sampling methods, using the *Prolific* platform (www.Prolific.co). Briefly, *Prolific* is an online data collection platform where researchers can advertise their studies to *Prolific* users who meet pre-defined study eligibility criteria. The platform requires participants to undertake in-depth and comprehensive vetting processes at initial sign-up, and periodically, to maximise the quality and authenticity of data. Past research comparing online data collection platforms (e.g., *Prolific, Qualtrics, MTurk*) has found that *Prolific* provides the highest-quality data with regard to the provision of meaningful answers, attention checks, followed instructions, and retained information [28]. In addition to the quality checks imposed by the platform itself, we also included two attention check questions within our survey. Participants who failed either attention check question, were excluded from our final sample of 588 participants.

### Measures

Survey items were adapted from PytlikZillig, Hamm [17], who provided a quantitative examination of people’s perceptions of trust across four institutional contexts. We measured 11 of the same dimensions relevant to trust as tested in this prior research. These were *loyal trust*, *perceived competence*, *perceived legitimacy*, *shared values*, *perceived care*, *perceived voice*, *perceived honesty*, *perceived fairness*, *cynical beliefs*, and *perceived bias*. We measured institutional trust in the police using 44-items (α = .98). We measured *limited* interpersonal trust (i.e., trust in someone with whom the respondent has a close relationships) using a subset of 30 of the most relevant items based on the institutional trust items (α = .96). For interpersonal trust, we asked participants to envision and name a person whom they knew well. From the provision of this name at the start of the survey, the 30 interpersonal trust items included the name of that person (e.g., “*I trust* [the specified name]”). This full list of items for both contexts are presented in Table 1. Internal consistency for these dimensions was good for each dimension with three or more items. As anticipated, dimensions including two items reported lower levels of internal consistency but were still in acceptable ranges for research purposes (see Table 1).

**Table 1:** Items Used to Measure 12 Dimensions of Trust in Institutional and Interpersonal Trust Contexts (*N* = 200).

Dimension	Label	Institutional Trust	Interpersonal Trust
Direct/Unspecified Trust	DU1	My confidence in my local police department is high	I have confidence in NAME
(α = .95;.88)	DU2	I have confidence in my local police department to do its job	I trust NAME
	DU3	I trust my local police department	
	DU4	I trust my local police department to do its job well	
Loyal Trust (α = .86;.83)	LO1	I have respect for my local police department representatives, even when I disagree with decisions they make	I have respect for NAME, even when I disagree with the actions they make
	LO2	I feel a sense of loyalty to my local police department	I feel a sense of loyalty to NAME
	LO3	I generally support my local police department, even when I disagree with some of its actions	I generally support NAME, even when I disagree with some of the actions they make
Perceived Competence (α = .92;.84)	CO1	Most decision makers at my local police department are competent to do their jobs	NAME is competent
	CO2	Most decision makers at my local police department are suitably qualified individuals	NAME is good at what they do
	CO3	Most representatives of my local police department have the knowledge they need to do their jobs	
	CO4	Overall, my local police department do a good job	
Perceived Legitimacy	LE1	My local police department is a legitimate organisation	NAME does the right thing
(α = .89;.85)	LE2	My local police department is a valid source of information	NAME is a valid source of information
	LE3	Members of my local police department are chosen through a fair process	NAME uses their power appropriately
	LE4	The procedures followed by my local police department are lawful	
	LE5	My local police department uses its power appropriately	
	LE6	My local police department is a legitimate law enforcement authority	
Shared Values (α = .94;.90)	SV1	I believe that my local police department shares my values toward justice	I believe that I share NAME’s values
	SV2	I share values of my local police department	NAME shares my values
	SV3	I believe that my local police department supports my values when it makes decisions	
	SV4	To the extent that I understand them, I share my local police department values regarding the future of my community	
	SV5	I share my local police department values about how they should do their job	
Perceived Care (α = .92;.84)	CA1	My local police department have the community’s best interests in mind when making decisions	NAME keeps me in mind when making decisions
	CA2	Most members of my local police department care about the residents that live in the areas that they work	NAME puts aside their own interests to make decisions that are right for me
	CA3	My local police department care about how the policies they make will impact residents	NAME cares about how the decisions they make will impact me
	CA4	My local police department puts aside its own interests to make decisions that are right for the community	
	CA5	For the most part, the decisions made by my local police department are made out of care and concern for community members	
Perceived Voice (α = .84;.66)	V1	I feel like my local police department listen to the opinions of the community	NAME listens to my opinions
	V2	Residents have a great say in important decisions that affect my local police department.	I can influence NAME’s decisions
	V3	Citizens can influence the decisions made by my local police department	NAME listens to the opinions of others
	V4	My local police department would listen to my opinions	
Perceived Honesty (α = .88;.89)	H1	My local police department is made up of mostly honest individuals	NAME is honest
	H2	Most officials in my local police department act with integrity	NAME has integrity
	H3	Even when it is difficult, my local police department still upholds its values	Even when it is difficult, NAME upholds their values
Perceived Fairness (α = .86;.87)	F1	I think that my local police department uses fair procedures to make its decisions	I think that NAME uses fair procedures to make decisions
	F2	My local police department has been fair in its dealings with the community	I feel that I have been treated fairly by NAME
	F3	In general, I feel that I have been treated fairly by my local police department	I think that NAME is fair in how they deal with others
Cynical Beliefs (α = .81;.76)	CB1	My local police department is out of touch with what is happening in the community	NAME is out of touch with what is happening with me
	CB2	My local police department does not protect my interests	NAME does not protect my interests
	CB3	My local police department use their power to try and control people like me	NAME uses their power to try and control me
	CB4	My local police department does not represent the community	
Perceived Bias (α = .77; 60)	B1	I think that my local police department act in the interests of some groups over others	I think that NAME acts in the interests of some groups over others
	B2	The decisions made by my local police department are biased	The decisions made by NAME are biased
	B3	My local police department is overly influenced by special interest groups	NAME is overly influenced by other people around them

Note. Cronbach’s alphas are presented for institutional and interpersonal trust, respectively.

### Procedure

The University of Western Australian Human Research Ethics Committee approved the study protocol (approval: 2021/ET000912). The study was distributed on the *Prolific* platform, with users who were current residents of Australia or New Zealand, proficient in the English language, and over 18 years of age eligible for participation (meaning that *Prolific* users who did not meet these eligibility criteria were unable to see the study). Prospective participants were then given a link to a digital participant information sheet and informed consent form. Participants provided their informed consent upon review of the information sheet in written (digital) format). Consenting participants were then redirected to complete the survey, hosted on the Qualtrics platform (www.Qualtrics.com). We included two attention check items to further verify participant attention (i.e., “*Please select ‘Disagree’ to this statement*”); any incorrect responses to this statement we deemed as invalid completion, and we excluded that participant data from the study. The participants were presented with either the institutional or interpersonal context items in counterbalanced order, with the order of individual items also counterbalanced to eliminate any systematic order-effects. Data was collected in two waves of collection using identical recruitment methods. The first wave (*n* = 387) occurred between November 1^st^ (2022) and February 11^th^ (2023). The second wave (*n* = 201) was collected between March 18^th^ (2024) and April 18^th^ (2024) and was collected to ensure the study had appropriate statistical power. Participants who completed the first intake were ineligible for the second intake.

Basic demographic characteristics (age, gender, level of education) were also collected before the completion of the survey. The average response time to complete this study was 9–10 minutes. All participants were compensated approximately $1.67 USD upon valid completion of the study.

### Analysis plan

All analyses were completed using Version 8.1.8.11 of Mplus [29]. Data was first screened and cleaned so that any incomplete or invalid response (e.g., those who failed attention check questions) were removed prior to the analyses. Measured items were treated as continuous variables and used as indicators for latent factors for all the models. The analyses can be broadly grouped into three phases.

***Phase one: First-order models:*** The first phase involved comparing a series of seven first-order CFAs derived from PytlikZillig, Hamm [17] where the items administered to the current sample were adapted. These first-order CFAs (various iterations of Fig 1b) moved from a simple one-factor model to the most complex many-factor model (see Table 2).

**Table 2:** Conceptual measurement models of trust and trust-relevant constructs in the local police department compared in the current study (*N* = 588).

		First-order Models						
Trust Constructs as Factors			Trust Constructs Combined into Factors					
Higher-Order and Bifactor Models		MF: Many-Factor Constructs	5F: Five-Factor	4FA: Four-Factor, Ability/Warmth	4FB: Four Factor, Positive/Negative	3F: Three Factor	2F: Two-Factor	1F: Compact Model
General factor	Direct/Unspecified Trust	Direct/Unspecified Trust	Trust	Trust	Trust	Trust	Trust	Trust
	Loyal Trust	Loyal Trust						
	Perceived Competence	Perceived Competence	Perceived Ability	Perceived Ability	Perceived Ability	Positive Attitudes	Perceived Trustworthiness	
	Perceived Legitimacy	Perceived Legitimacy						
	Perceived Care	Perceived Care	Perceived Benevolence	Perceived Benevolence	Perceived Warmth			
	Perceived Voice	Perceived Voice						
	Perceived Honesty	Perceived Honesty	Perceived Integrity	Perceived Integrity				
	Perceived Fairness	Perceived Fairness						
	Perceived Shared Values	Perceived Shared Values	Values/Identification					
	Cynical Values	Cynical Values				Negative Attitudes		
	Perceived Bias	Perceived Bias	Perceived Integrity					

Note. Many Factor (MF) Constructs model treats each dimension as a factor. Other models included combinations of the constructs separated in the MF and Higher-Order MF model. Models are presented in a way that ranges from most complex (i.e., the most parameters) to the least complex (i.e., fewer parameters).

Goodness of fit statistics were compared to conventional cut-offs by Hu and Bentler (1999), and between each model. To account for the non-normal distribution of the trust items, robust maximum likelihood (MLR) rotation was used when assessing model fit. The test of log likelihood was used to compare whether a model with more parameters (the *alternative* model with more free parameters) produced *significantly* improved fit to the preceding model with fewer parameters (the *null* model). A statistically significant test of log likelihood suggests that the alternative model is produces significantly better fit to the null model.

***Phase two: Higher-order and bi-factor models.*** The second phase involved comparing the best-fitting first-order model (identified in phase one) to two models that introduce a general factor. These are (a) a higher-order CFA (Fig 1c), and (b) a bi-factor CFA (Fig 1d). Consistent with phase one, the comparison of fit statistics and log likelihood test was enlisted to compare the fit of the models.

***Phase three: Exploratory structural equation modelling (ESEM).*** The final phase integrated ESEM to further explore the relationship between trust dimensions and the items used to measure both institutional and interpersonal dimensions of trust, respectively. Using the a priori factor structure of the best-fitting first-order model from phase one, and the higher-order and bi-factor models in phase two, item cross-loadings were permitted (unlike the prior phases) but were targeted to be close to zero. We used the de Beer and van Zyl [30] ESEM tool, which provides an intuitive and efficient method to create the necessary syntaxes to perform the ESEM (Fig 1e), higher-order ESEM (Fig 1f), and bi-factor ESEM (Fig 1g) in Mplus. The tool uses the Asparouhov and Muthén [31] ESEM estimation procedure, with a clear process in producing, reporting, and comparing competing ESEM models recently published by van Zyl and ten Klooster [32]. Obtained model fit statistics allowed the fit of these models to be statistically compared to the many-factor CFA model (given that this was a nested model) using the log likelihood test. Comparison of the obtained model fit statistics were used to compare which of the ESEM models produced the best fit. The results for institutional trust and reported together, before the results for interpersonal trust.

## Results

### Models of institutional trust

***Phase one: First-order CFAs.*** The fit statistics for the seven CFAs of first-order models of institutional trust where the factor structure and items were derived from PytlikZillig, Hamm [17] revealed that the many-factor model produced the best fit statistics. These fit statistics are reported as part of Table 3. Log-likelihood tests were performed to compare each increasingly complex first-order model to the preceding model (i.e., the two-factor CFA was compared to the one-factor CFA, and so on). Each of these tests were statistically significant, suggesting that the alternative (i.e., more complex model) resulted in a significantly improved model fit. This was further supported by the obtained model fit statistics, with the many-factor model reporting better CFI, TLI, RMSEA, SRMR, AIC, and sample-adjusted BIC (aBIC) values (see Table 3). Thus, of the first-order models of institutional trust, the many-factor model produced the best fit to the data.

**Table 3:** Goodness of fit statistics for competing models of dimensions of institutional trust dimensions (*N* = 588).

	Fit Statistics									Log likelihood tests ^c^				
Models	χ^2^	*df*	χ^2^/*df*	CFI	TLI	RMSEA (90% CI)	SRMR	AIC	aBIC	LL	SCF	# Free Parameters	Test statistic	LL test sig.
*Conventional Indicators of good fit*	–	–	smaller value = better fit	≥.95	≥.95	≤.06	≤.05	smaller value = better fit		–	–	–	–	–
**First-Order Models**	**χ** ^**2**^	***df***	**χ** ^**2**^ **/*df***	**CFI**	**TLI**	**RMSEA (90% CI)**	**SRMR**	**AIC**	**aBIC**	**LL**	**SCF**	**# Free Parameters**	**Test statistic**	**LL test sig.**
1F: *Compact Model*	2915.908	902	3.23	0.894	0.889	0.062 (0.059, 0.064)	0.044	68328.564	68487.237	−34032.28	1.3445	132	–	–
2F: *Personal Trust, and Personal Trustworthiness*	2821.059	901	3.13	0.899	0.894	0.06 (0.058, 0.063)	0.044	68205.631	68365.506	−33969.82	1.3535	133	80.52	<.001
3FA: *Personal Trust, Perceived Ability, and Perceived Warmth*	2739.861	899	3.05	0.903	0.898	0.059 (0.056, 0.062)	0.044	68099.11	68261.389	−33914.56	1.3696	135	37.62	<.001
3FB: *Personal Trust, Positive Attitudes, and Negative Attitudes*	2490.753	899	2.77	0.916	0.912	0.055 (0.052, 0.057)	0.041	67784.489	67946.768	−33757.25	1.364	135	161.11	<.001
4F: *Personal Trust, Perceived Ability, Perceived Benevolence, Perceived Integrity*	2617.245	896	2.92	0.909	0.904	0.057 (0.055, 0.06)	0.043	67946.407	68112.292	−33835.20	1.3744	138	81.65	<.001
5F: I*nstitutional trust and ability/ benevolence/integrity/values (ABIV)*	2537.245	892	2.84	0.913	0.908	0.056 (0.053, 0.059)	0.044	67846.346	68017.04	−33781.17	1.3874	142	66.05	<.001
MF: *Many-factor model*	1776.299	847	2.10	0.951	0.945	0.043 (0.04, 0.046)	0.038	66956.332	67181.118	−33291.17	1.3978	187	683.63	<.001
**Higher-Order Model**	**χ** ^**2**^	***df***	**χ** ^**2**^ **/*df***	**CFI**	**TLI**	**RMSEA (90% CI)**	**SRMR**	**AIC**	**aBIC**	**LL**	**SCF**	**# Free Parameters**	**Test statistic**	**LL test sig.**
TF: *Many-factor model with a general factor*	2086.398	891	2.34	0.937	0.933	0.048 (0.045, 0.05)	0.041	67265.165	67437.061	−33489.58	1.4213	143	301.52^b^	<.001
**Bifactor Model**	**χ** ^**2**^	***df***	**χ** ^**2**^ **/*df***	**CFI**	**TLI**	**RMSEA (90% CI)**	**SRMR**	**AIC**	**aBIC**	**LL**	**SCF**	**# Free Parameters**	**Test statistic**	**LL test sig.**
BF: *Bifactor model with a general factor*	2111.00	870	2.43	0.935	0.929	0.049 (0.047, 0.052)	0.121	67324.655	67521.794	−33498.33	1.435	164	372.3^b^	<.001
**Exploratory Structural Equation Models (ESEMs)**	**χ** ^**2**^	***df***	**χ** ^**2**^ **/*df***	**CFI**	**TLI**	**RMSEA (90% CI)**	**SRMR**	**AIC**	**aBIC**	**LL**	**SCF**	**# Free Parameters**	**Test statistic**	**LL test sig.**
TFESEM: *Many-factor model with a general factor ESEM* (i.e., higher-order ESEM)	670.31	561	1.19	0.994	0.990	0.018 (0.012, 0.023)	0.014	66038.923	66607.502	−32546.46	1.4854	473	966.28^b^	<.001
MFESEM: *Many-factor ESEM*	660.07	517	1.28	0.992	0.986	0.022 (0.016, 0.026)	0.010	66053.848	66675.318	−32509.92	1.5472	517	937.09^b^	<.001
BFESEM: *Bifactor model with a general factor ESEM* (i.e., bifactor ESEM)	599.981	484	1.24	0.994	0.988	0.02 (0.014, 0.025)	0.009	66045.270	66706.408	−32472.64	1.535	550	1019.72^b^	<.001

*Note*. *N* = 200. χ^2 ^= Chi-square. *df* = degrees of freedom. CFI = Comparative Fit Index. TLI = Tucker-Lewis Index. RMSEA = Root Mean Square Error of Approximation. SRMR = Standardised Root Mean Square Residual. AIC = Akaike Information Criterion. BIC = Bayesian Information Criterion. LL = log likelihood. Values in bold depict the most well-fitting value of those observed. Recommended cut-off values as reported in previous work (Hu & Bentler, 1999). ^a^ Test statistic derived when comparing this model to the best fitting three-factor model. ^b^ In this comparison, the many-factor model specified as the alternative model. ^c^ Unless specified, each log likelihood test reported is the result of comparing the model to the model reported in the row above.

***Phase two: Higher-order and bi-factor CFAs***. The higher-order and bi-factor models both reported adequate fit statistics when benchmarked against conventional model fit statistics. Moreover, model fit statistics revealed that higher-order model had slightly better fit than the bi-factor model. The fit statistics for the higher-order and bi-factor CFAs were compared with the many-factor model (from the first-order CFAs). When inspecting the model fit statistics and log likelihood tests, the results suggested that the many-factor model produced significantly better fit than the higher-order and bi-factor models (based on tests of the log-likelihood for the higher-order model, and fit statistics for the bi-factor model).

**Fig 2:**
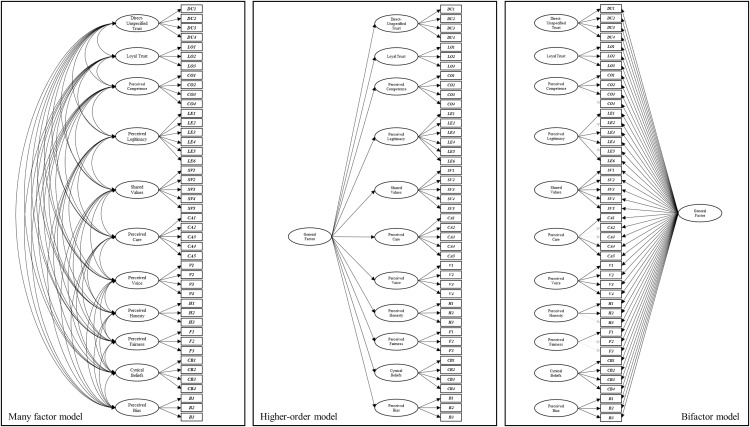
Visual representation of confirmatory factor analyses (CFAs) of the many-factor (left), higher-order (middle), and bi-factor (right) models of institutional trust. Significant pathways are depicted in bold, whereas non-significant pathways are dashed lines in grey.

Next, we inspected the pattern of factor loadings across the many factor, higher-order, and bi-factor models. In both the many-factor and higher-order models, all the specified pathways were significant. In the bi-factor model, the pattern of findings revealed that the general factor was indicated by each of the individual items. However, several items did not also have significant loadings onto the intended specific factor (two items from the *legitimacy* dimension, two items from the *care* dimension, one item from the *honesty* dimension, and two items from the *fairness* dimension; see Fig 2).

Thus, the general factor in the bi-factor model was able to capture most of the variance in these items, providing some evidence that the general trait is the primary dimension being captured by these items, as well as significant variance on the other items which also retained significant loadings on the specific factors. This pattern of findings suggested that there may be some additional utility in further investigating how items and factors would be related when flexible and exploratory procedures were enlisted.

**Fig 3:**
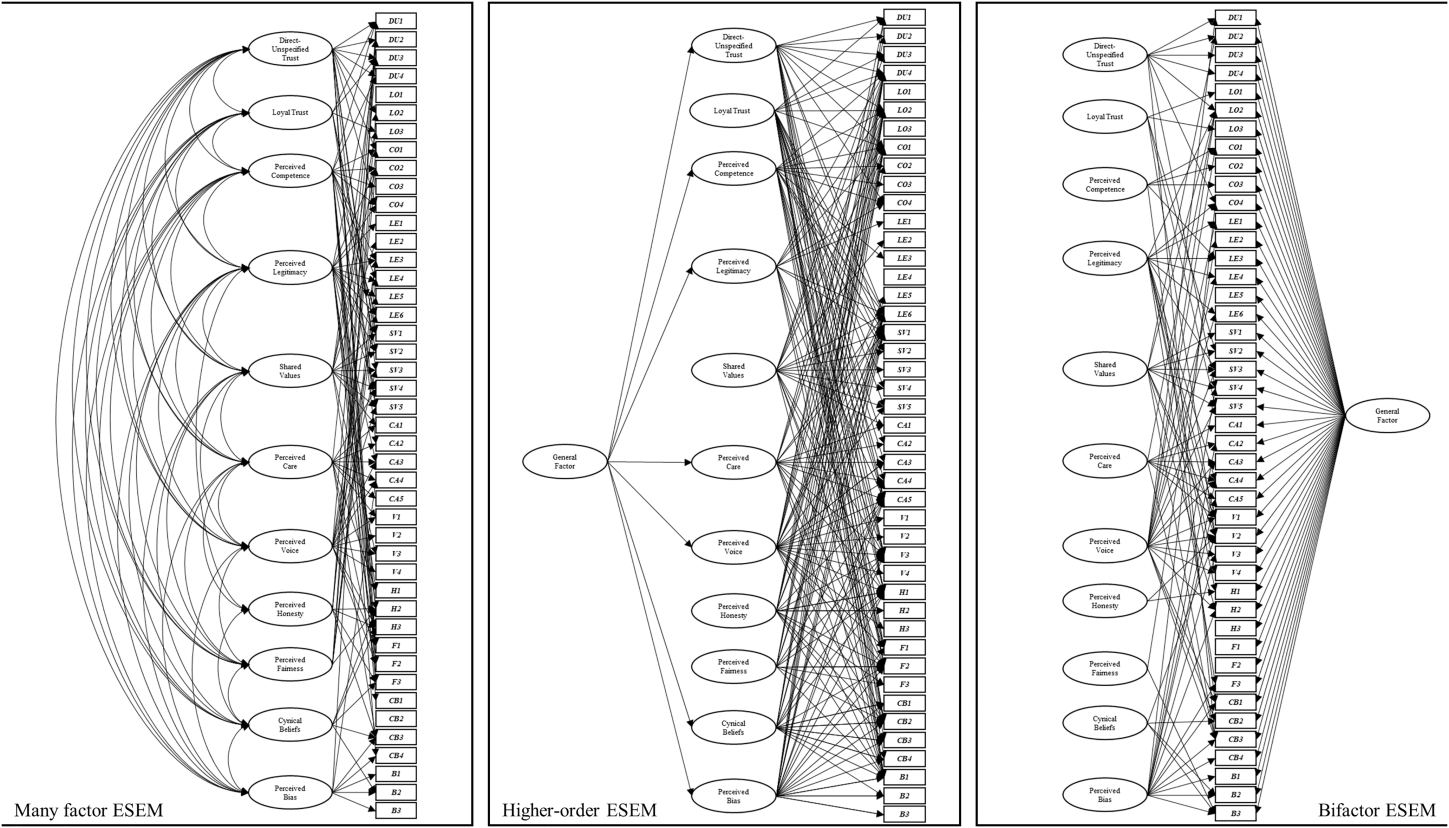
Visual representation of exploratory structural equation models (ESEMs) of the many-factor (left), higher-order (middle), and bi-factor (right) models of institutional trust. Only significant pathways are presented.

***Phase three: ESEMs of the many-factor, higher-order, and bi-factor models.*** Each of the ESEMs produced superior fit to the many-factor, higher-order, and bi-factor CFA models (see Fig 3).

Moreover, they also provided excellent fit statistics based on conventionally acceptable cut-offs. This finding was anticipated, considering the increased number of free parameters and opportunity for item cross-loadings that were not permitted in the first-order, higher-order, and bi-factor CFAs. In observing the model fit statistics, the higher-order ESEM provided slightly better fit, followed by the bi-factor ESEM, and then the many-factor ESEM. However, a key advantage of the ESEM approach is to explore how the pattern of loadings may differ to the fixed pattern of loadings specified in CFAs. The loading pattern revealed mixed findings.

In the higher-order ESEM, the higher-order general factor was no longer indicated the by the *shared values, honesty,* and *fairness* dimensions. There were also many item cross-loadings, with very few items being indicated by only one, and/or the intended specific factor. The bi-factor model was also characterised by similar cross-loadings onto specific factors. However, a key finding here was that, even when specific factors were able to be identified by any item, the general factor in the bi-factor model was indicated by all items. The many factor ESEM was also characterised by a similar pattern of item cross-loadings, which demonstrated that the specific factors may be less distinct and that a general factor explains much of the variance in these items.

### Models of interpersonal trust

***Phase one: First-order CFAs.*** The fit statistics for the seven CFAs of first-order models of interpersonal trust derived from PytlikZillig, Hamm [17] revealed that the many-factor model produced the best fit statistics (see Table 4). These results mirror those observed with institutional trust, as the fit of the many-factor model was significantly better fit compared to each preceding model (evidenced by the log-likelihood tests and model fit statistics). Thus, of the first-order models of interpersonal trust, the many-factor model produced the best fit.

**Table 4:** Goodness of fit statistics for competing models of dimensions of limited interpersonal trust dimensions (*N* = 588).

	Fit Statistics									Log likelihood tests ^c^				
Models	χ^2^	*df*	χ^2^/*df*	CFI	TLI	RMSEA (90% CI)	SRMR	AIC	aBIC	LL	SCF	# Free Parameters	Test statistic	LL test sig.
*Conventional Indicators of good fit*	–	–	smaller value = better fit	≥.95	≥.95	≤.06	≤.05	smaller value = better fit		–	–	–	–	–
**First-Order Models**	**χ** ^**2**^	***df***	**χ** ^**2**^ **/*df***	**CFI**	**TLI**	**RMSEA (90% CI)**	**SRMR**	**AIC**	**aBIC**	**LL**	**SCF**	**# Free Parameters**	**Test statistic**	**LL test sig.**
1F: *Compact Model*	1582.472	405	3.907338	0.876	0.867	0.070 (0.067, 0.074)	0.051	47093.28	47201.47	−23456.6	1.7121	90	–	–
2F: *Personal Trust, and Personal Trustworthiness*	1525.633	404	3.776319	0.882	0.873	0.069 (0.065, 0.072)	0.051	47008.58	47117.97	−23413.3	1.7363	91	22.16	<.001
3FA: *Personal Trust, Perceived Ability, and Perceived Warmth*	1512.268	402	3.761861	0.883	0.873	0.069 (0.065, 0.072)	0.050	46989.41	47101.21	−23401.7	1.7426	93	11.40	.003
3FB: *Personal Trust, Positive Attitudes, and Negative Attitudes*	1341.461	402	3.336968	0.901	0.893	0.063 (0.059, 0.067)	0.049	46765.47	46877.26	−23289.7	1.7237	93	379.58	<.001
4F: *Personal Trust, Perceived Ability, Perceived Benevolence, Perceived Integrity*	1452.986	399	3.641569	0.889	0.879	0.067 (0.063, 0.071)	0.050	46915.56	47030.95	−23361.8	1.7305	96	74.22 ^a^	<. 001
5F: I*nstitutional trust and ability/ benevolence/integrity/values (ABIV)*	1387.183	395	3.511856	0.895	0.885	0.065 (0.062, 0.069)	0.052	46827.49	46947.7	−23313.7	1.7365	100	51.080	<.001
MF: *Many-factor model*	777.67	350	2.221914	0.955	0.944	0.046 (0.041, 0.05)	0.038	46071.54	46245.84	−22890.8	1.704	145	518.46	<.001
**Higher-Order Model**	**χ** ^**2**^	***df***	**χ** ^**2**^ **/*df***	**CFI**	**TLI**	**RMSEA (90% CI)**	**SRMR**	**AIC**	**aBIC**	**LL**	**SCF**	**# Free Parameters**	**Test statistic**	**LL test sig.**
TF: *Many-factor model with a general factor*	993.421	394	2.521373	0.937	0.930	0.051 (0.047, 0.055)	0.045	46285.13	46406.54	−23041.6	1.7982	101	202.68^b^	<.001
**Bifactor Model**	**χ** ^**2**^	***df***	**χ** ^**2**^ **/*df***	**CFI**	**TLI**	**RMSEA (90% CI)**	**SRMR**	**AIC**	**aBIC**	**LL**	**SCF**	**# Free Parameters**	**Test statistic**	**LL test sig.**
BF: *Bifactor model with a general factor*	930.032	387	2.403183	0.943	0.936	0.049 (0.045, 0.053)	0.04	46215.88	46345.7	−22999.9	1.765	108	143.04 ^b^	<.001
**Exploratory Structural Equation Models (ESEMs)**	**χ** ^**2**^	***df***	**χ** ^**2**^ **/*df***	**CFI**	**TLI**	**RMSEA (90% CI)**	**SRMR**	**AIC**	**aBIC**	**LL**	**SCF**	**# Free Parameters**	**Test statistic**	**LL test sig.**
TFESEM: *Many-factor model with a general factor ESEM* (i.e., higher-order ESEM)	266.477	204	1.30626	0.993	0.986	0.023 (0.014, 0.030)	0.016	45660.95	46010.75	−22539.5	1.5993	291	467.48^b^	<.001
MFESEM: *Many-factor ESEM*	239.267	160	1.495419	0.992	0.977	0.029 (0.021, 0.036)	0.010	45667.74	46070.43	−22498.9	1.641	335	492.14^b^	<.001
BFESEM: *Bifactor model with a general factor ESEM* (i.e., bifactor ESEM)	238.54	141	1.691773	0.99	0.968	0.034 (0.027, 0.042)	0.009	45674.72	46100.26	−22483.4	1.655	354	501.33	<.001

*Note*. *N* = 200. χ^2 ^= Chi-square. *df* = degrees of freedom. CFI = Comparative Fit Index. TLI = Tucker-Lewis Index. RMSEA = Root Mean Square Error of Approximation. SRMR = Standardised Root Mean Square Residual. AIC = Akaike Information Criterion. BIC = Bayesian Information Criterion. Values in bold depict the most well-fitting value of those observed. Recommended cut-off values as reported in Hu and Bentler (1999). ^a^ Test statistic derived when comparing this model to the best fitting three-factor model. ^b^ In this comparison, the many-factor model specified as the alternative model. ^c^ Unless specified, each log likelihood test reported is the result of comparing the model to the model reported in the row above.

**Fig 4:**
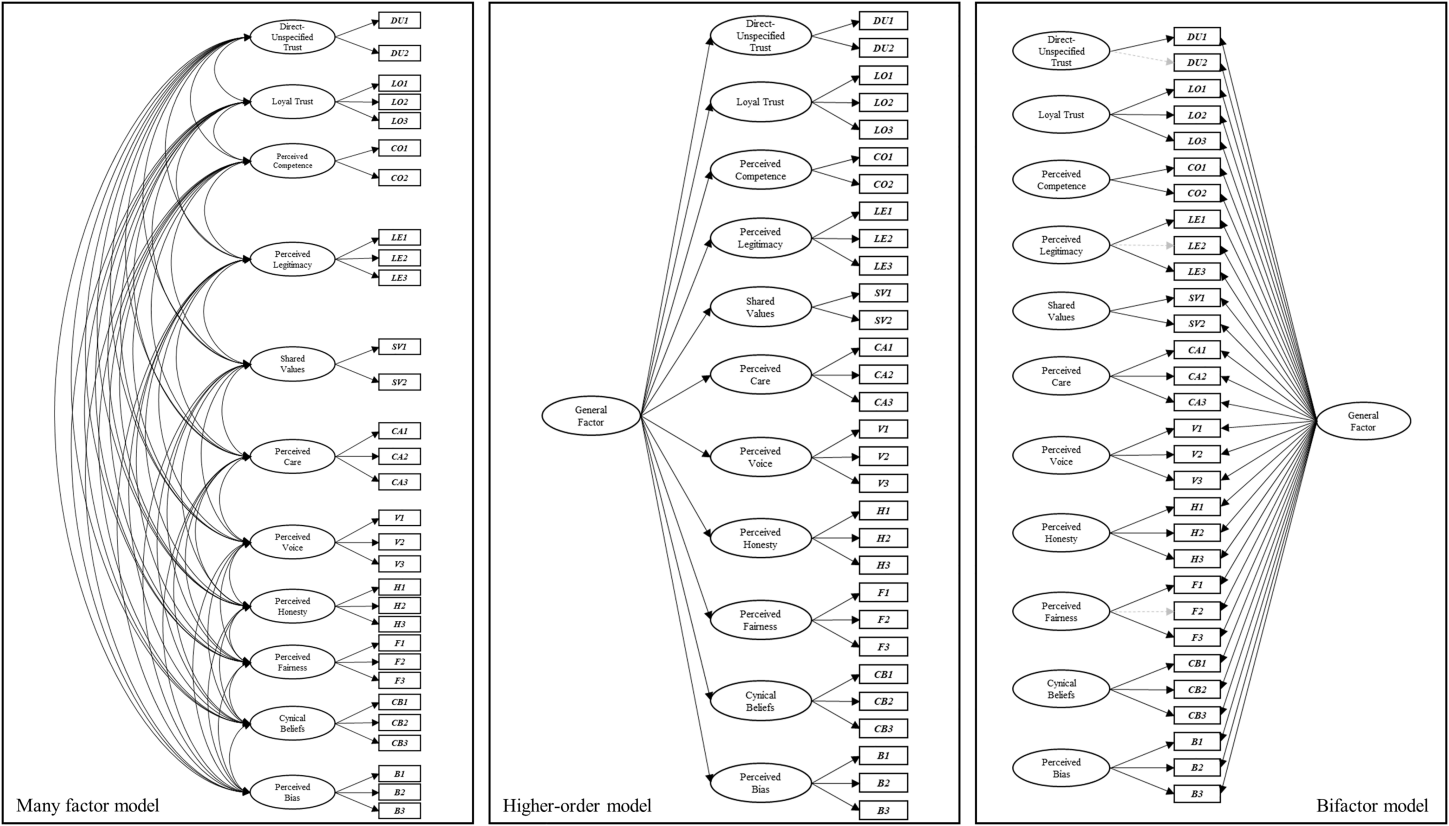
Visual representation of confirmatory factor analyses (CFAs) of the many-factor (left), higher-order (middle), and bi-factor (right) models of interpersonal trust. Significant pathways are depicted in bold, whereas non-significant pathways are dashed lines in grey.

***Phase two: Higher-order and bi-factor CFAs***. The higher-order and bi-factor models both produced good fit statistics when benchmarked against conventional model fit statistics. Unlike the observations made in the institutional trust items, the fit statistics revealed that the bi-factor model had slightly better fit than the higher-order model (see Fig 4). The fit statistics for the higher-order and bi-factor CFAs were compared with the many-factor model (from the first-order CFA). Like the institutional trust items, the many-factor model produced significantly better fit than the higher-order and bi-factor models (based on tests of the log-likelihood for the higher-order model, and fit statistics for the bi-factor model).

Next, we inspected the pattern of factor loadings across the many-factor, higher-order, and bi-factor models. In both the many-factor and higher-order models, all the specified pathways were significant. In the bi-factor model, the pattern of findings revealed that the general factor was indicated by each of the individual items. However, three items no longer had loadings onto the intended specific factor (one from the *direct/unspecified* trust dimension, one from the *legitimacy* dimension, and one from the *fairness* dimension).

**Fig 5:**
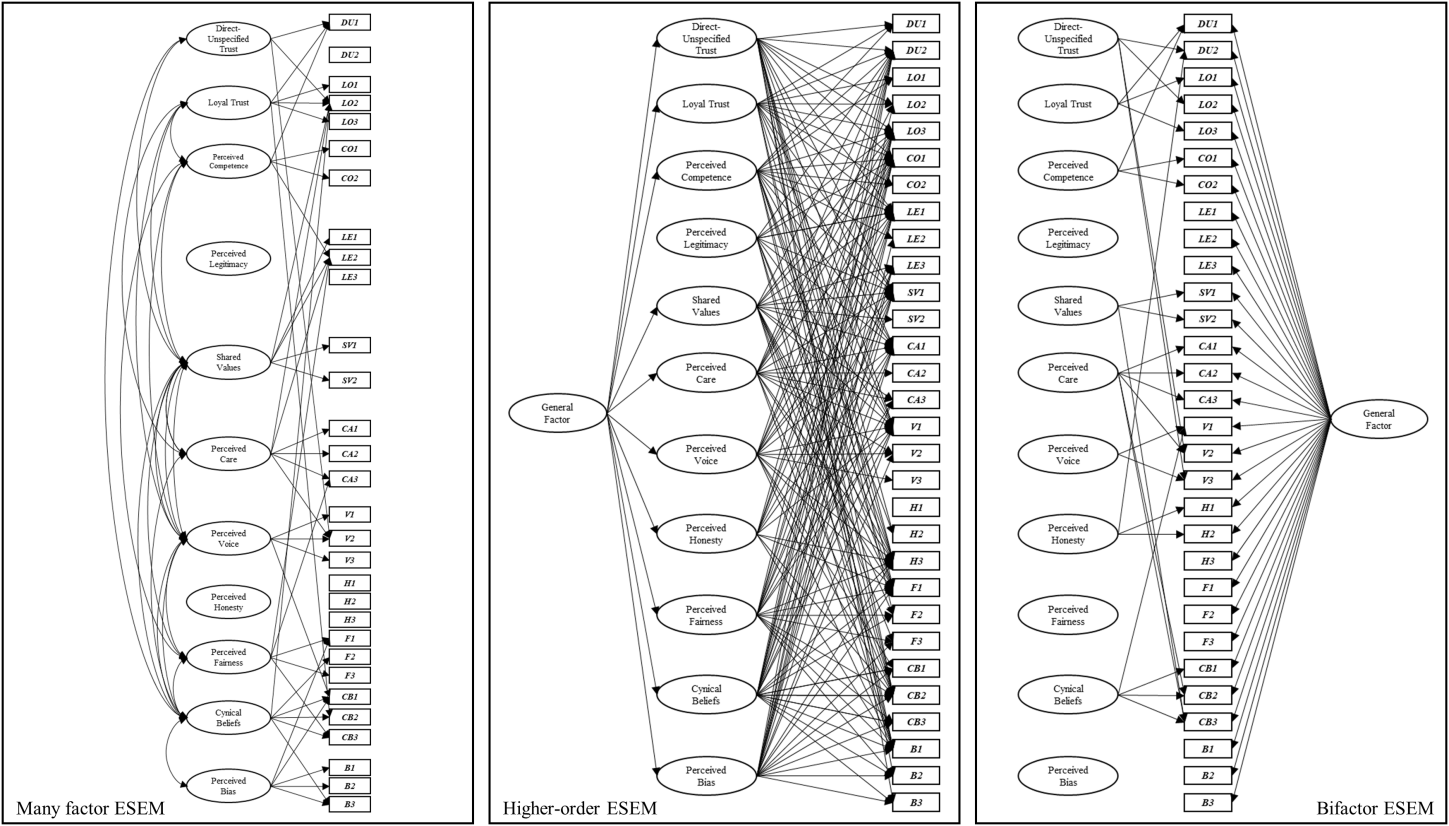
Visual representation of exploratory structural equation models (ESEMs) of the many-factor (left), higher-order (middle), and bi-factor (right) models of interpersonal trust. Only significant pathways are presented.

***Phase three: ESEMs of the many-factor, higher-order, and bi-factor models.*** Each of the ESEMs expectedly produced superior fit to the many-factor, higher-order, and bi-factor CFA models, and excellent fit statistics based on conventionally acceptable cut-offs. Consistent with the institutional trust models, the higher-order ESEM provided slightly better fit, followed by the bi-factor ESEM, and then the many-factor ESEM. However, as was the case for the institutional trust ESEMs, the pattern of item loadings on specific and general factors was prioritised as a method to advance our understanding on the relationship between trust items and dimensions (see Fig 5).

In the higher-order ESEM, the higher-order general factor was no longer indicated the by the *legitimacy*. There were also many item cross-loadings, with very few items being indicated by only one, and/or the intended specific factor. The results highlight a high degree in overlap between specific factors.

The bi-factor model for interpersonal trust was populated with fewer specific factor cross-loadings than the higher-order model of interpersonal trust, and the comparable bi-factor model of institutional trust previously examined. Several item cross-loadings were also identified. However, like the institutional trust bi-factor ESEM, all items were found to have specific loadings onto the general factor. Moreover, many of the specific factors (*legitimacy, fairness,* and *perceived bias*) could not account for any variance across *any* of the 30 items assessed beyond which could already be explained by the general factor. The results here suggest that, for interpersonal trust, and like institutional trust, a general factor in the bi-factor model was the only latent factor to consistently explain variance across all trust items.

## Discussion

The aim of this study was to explore the latent factor structure of a previously developed self-report survey-based assessment of institutional trust [17] that was also adapted for use in an interpersonal context, by employing novel statistical approaches. In summary, first, we replicated CFA-based approaches to compare alternative latent factor structures. We then expanded upon earlier approaches by interrogating a potential *general* factor structure of trust – a construct that serves to underpin the variance between specific factors and observed items. Finally, we enlisted the use of bi-factor and ESEM methods, two emerging approaches to scale development that have not readily been utilized in trust research.

Within the parameters of the current study’s strengths and limitations, our results may have important implications for the conceptualisation and dimensionality of trust, as well as for institutions or individuals hoping to cultivate trust. These are discussed below.

### Many-factor models provide the best fitting first-order models of trust

The many-factor correlated first-order model for both institutional and interpersonal trust produced the best fit to the data compared to other first-order models, supporting hypothesis H1. Across both the institutional and interpersonal contexts, the CFA many-factor first-order models of trust that comprised 11-dimensions (see Fig 2 and Fig 4) produced a better fit when compared with six alternative model conceptualizations (see Table 2). These alternative conceptualizations were more parsimonious than the best-fitting many-factor model, as multiple trust dimensions were grouped into factors. For example, the *five-factor* model grouped *perceived care* and *perceived voice* into a factor named *perceived benevolence*. However, similar to PytlikZillig, Hamm’s [17] assessment of institutional trust, the more parsimonious first-order models did not produce better fit for items measuring institutional trust or interpersonal trust in the present study. If relying on analyses comparing first-order models, these findings would suggest that an optimal way to consider the associations between trust-relevant dimensions is as a set of distinct, yet correlated dimensions.

For the ESEM many-factor models, in both the institutional and interpersonal contexts (see Fig 3 and Fig 5), variance in most items was explained by several specific factors. However, we also identified that for interpersonal trust, there were several items that did not have *any* variance explained by the specific factors, and there were two specific factors with no significant item loadings (*perceived legitimacy* and *perceived honesty*). One interpretation of these findings is that items purportedly assessing a specific factor (*perceived legitimacy* or *perceived honesty*) may, statistically, be better explained as assessing a different factor. Alternatively, these results could be interpreted as highlighting the complex interrelationships between trust items and dimensions, providing support for applying models that can better accommodate statistical overlap between specific factors and observed items, such as higher-order and bi-factor ESEMs. Whether first-order models would remain the best way to conceptualize trust-related dimensions was tested in comparison to higher-order and bi-factor models.

### Higher-order models of trust offer a good fit, and some conceptual advantages

Across both the institutional and interpersonal context, the fit statistics of the higher-order models (CFA and ESEM) provided a very good fit to the data, supporting hypothesis H2. The higher-order models produced model fit statistics that rivalled the corresponding many-factor first-order models, though as observed in Hamm and Hoffman [19], they were often slightly outperformed by the first-order models. Consistent with Hamm and Hoffman [19], we argue that decisions concerning which model structure is ‘preferred’ when faced with similarly suitable model fit statistics is a decision that cannot be based on statistics alone. Instead, decision making processes should also be guided by a strong theoretical basis. In their study, Hamm and Hoffman [19] settled on the higher-order model as superior, pointing to the expectation of slightly poorer fit due to the more complex nature of the model, negligible difference in model fit values, and evidence that higher-order factor scores had unique predictive utility in explaining outcomes thought to be fostered by trust.

Support for higher-order models of other human phenomena (e.g., intelligence, or psychopathology) has helped advance knowledge within these respective fields for several decades. The trust literature and the psychopathology literature share similarities in that (a) they acknowledge the existence of ‘dimensions’ of phenomena that are neither identical nor entirely independent, (b) people who demonstrate elevated traits in one dimension often demonstrate elevated traits in others, indicating correlations between dimensions, and (c) treatment or intervention efforts targeting one dimension may yield changes to non-targeted dimensions. As a trust-based example, it could be argued that someone who perceives that their peer is *caring* might also perceive them to be *fair* and *honest*. Indeed, this was reflected in the strong positive correlations between the dimensions in our study, and offered preliminary empirical support for the assertion that a general factor could further explain how trust items and dimensions are associated.

To guide our interpretations of the empirical foundation, our thinking was informed by the theoretical perspective of epistemic trust [33], which we saw as a construct that could align with the notion of a general latent factor. Before embarking on a discussion of the implications of a general factor in relation to the higher-order model results, we discuss the insights offered by the bi-factor models derived from the CFAs and ESEMs, respectively.

### Bi-factor models of trust may challenge a priori associations between specific factors and indicators

To our knowledge, there has only been one previously published study that has tested a bi-factor model in a multi-dimensional assessment of trust that also produced support for a bi-factor conceptualisation [34]. Our findings expand upon this work by engaging with a larger battery of items spanning the contexts of institutional and interpersonal trust, arriving at a similar conclusion, whereby bi-factor models provided a good fit to the data and addressed some of the limitations associated with higher-order models.

Bi-factor models position the general factor as a construct that accounts for the common variance across all items, and the specific factors that account for the variance in the items that measure each (see Fig 2 and Fig 4). This differs from higher-order model formulations, which assume that the first-order dimensions are influenced by the higher-order factor, and that the general factor is not directly assessed by the items but ascertained via the influence it has on items via the first-order factors.

For trust researchers, we see several advantages offered by bi-factor models beyond model fit statistics. First, bi-factor models help absolve issues of multicollinearity [35]. We believe this is particularly relevant for the trust literature, where correlations between trust-related dimensions are often very high (e.g., *r* > .85 and above). Second, the separation of variance attributed to the general factor versus the specific factors also enhances measurement precision for each [36]. We recognised this in our study, where across both institutional and interpersonal contexts the pattern of item loadings after CFAs revealed that several items no longer loaded onto their specific factor once the general factor was included in the model.

Specifically, for institutional trust, items from the *perceived competence, perceived legitimacy, perceived care, perceived honesty,* and *perceived fairness* dimensions no longer loaded onto their respective specific factor once the general factor was included in the model (see Fig 2). For interpersonal trust, this occurred for items from the *direct-unspecified trust, perceived legitimacy,* and *perceived fairness* dimensions (see Fig 4). These results indicated that once items were free to load onto a general factor, the variance in these items was no longer explained by the a priori specific factor. In other words, these items may be a measure of the general construct rather than the specific factor (dimension) they were ascribed to, challenging the conceptual associations between these items and their associated trust dimension.

### Statistical interpretations of the exploratory structural equation models (ESEMs)

The ESEM versions of the many-factor, higher-order, and bi-factor models all produced excellent fit statistics, and fit statistics that exceeded their non-ESEM counterparts. This was expected, given the capacity for items to load onto any specific or general factors included in the model in ESEM and not just those they were ‘forced’ to in the CFAs. The utility of ESEM lies in their ability to examine the *pattern* of loadings of items and factors.

The results were not entirely consistent with hypothesis H3, as each ESEM was characterized by numerous item and factor cross-loadings (see Fig 3 and Fig 5). These made interpretability challenging, as there were very few examples of items that had variance explained by only their corresponding specific factor (as indicated in the CFAs). Additionally, some observed items were not indicated by *any* specific factor. The results of the ESEMs highlight potential shortcomings associated with the CFA approach to scale validation of trust items. Specifically, ‘forcing’ observed items to be indicators of *one* specific factor of trust may inadvertently perpetuate perceptions that these specific factors are both statistically and conceptually distinct.

For institutional trust, the higher-order ESEM revealed that the general factor did not explain variance in the dimensions of *loyal trust, shared values, perceived honesty,* or *perceived fairness*. For interpersonal trust, the higher-order model revealed that the general factor did not explain variance in the dimension of *perceived legitimacy*. This again highlighted a potential limitation regarding the way in which the observed items included in our study have previously been grouped into their respective *specific* factors based on past research. Variance in many of the items from our analysis appear to be indicated by other unintended specific factors. The ability of a general factor to explain variance in the observed items may also be attenuated as an artifact of the positioning of specific factors relative to the general factor and observed items. These results added weight to the rationale for a bi-factor ESEM approach to further interrogate the potential role of a general factor whilst limiting any statistical concerns brought on by a higher-order model.

The bi-factor ESEM models produced similar patterns of cross-loadings across specific factors, and several items with no loadings on any specific factor. For interpersonal trust, the bi-factor ESEM identified several specific factors that no longer explained variance in any observed items once the general factor was accounted for (*perceived bias*, *fairness*, and *legitimacy*). Across institutional and interpersonal trust contexts, the bi-factor ESEM demonstrated that a significant proportion of variance in each item was explained by a *general factor*. We consider this to be important, as even when items were free to load across all specific factors and the general factor, the general factor explained significant variance in each of the observed items, supporting its empirical relevance. It also represented evidence of a benefit of bi-factor approaches relative to higher-order approaches.

The bi-factor ESEM findings also presented support for a potentially novel method of calculating trust scores. Specifically, researchers could calculate a *factor* score for a person’s *‘general trust’,* derived from the unique variance in each item weighted to the general factor and not the specific factors. These scores could then be included in studies that seek to use this value as a potential predictor of future behaviour.

### Conceptual interpretations of the bi-factor ESEMs, and some implications

We recognize that the institutional and interpersonal contexts exist as mostly separate fields, and that there may be some utility in distinguishing potential differences between the trust processes in each. We also recognize that this study specifically measured *limited* interpersonal trust by asking people to rate their trust in *one* person whom they have a personal relationship with, and institutional trust by asking people to rate their trust in *one* institution, namely the police, an area that has spawned its own sub-discipline of investigation [37]. These two entities do not represent all people or institutions where trust is formed; thus, our interpretation is couched within this constraint.

In their daily lives, people must form judgements as to whether to trust a wide range of institutions. Our use of the police as the target institution in the current study aligns with prior theories and research. The police represent a visible and routinely encountered arm of the state, and one that the general public would generally consider acting in the public’s interest and behave with fairness and integrity. This visibility may help our sample to conjure a similar mental representation of the institution when compared to other, more ambiguous, options such as the government, or banks. Prior research has also shown that people’s trust in the police is correlated with broader measures of institutional trust, including trust in the government, the legal system, and civil institutions [38]. Experiences with high-salience institutions such as the police are known to shape people’s inferences about other, less-salient, institutions [39]. Accordingly, the police may serve as a proxy for broader institutional trust. However, as the scope of our current study does not permit us to assess this, the inclusion of other institutions in future studies would allow this to be further evaluated.

When comparing the rightmost diagrams in Fig 3 (institutional trust) and Fig 5 (interpersonal trust), a noticeable difference is the density of the cross-loadings across the specific dimensions. Specifically, the institutional trust dimensions have greater shared variance across the tested items. This indicated that, in a bi-factor ESEM, the specific factors of institutional trust may continue to explain variance in the observed trust items above and beyond a person’s general sense of trust (captured by the general factor). Though preliminary, we may tentatively attribute the importance of the dimensions to the proposition that, when forming trust-specific appraisals of an institution (e.g., *‘*the police’), in addition to a general sense of trust, people may draw upon experiences and influences more widely, compared to forming trust-specific appraisals of a known other. Our results suggest it may be possible for institutions to positively impact trust-specific appraisals made by members of the public via demonstrations of loyalty, competence, legitimacy, shared values, care, honesty, fairness, lack of cynicism and bias, and by providing opportunities for voice.

While specific trust dimensions also appeared to guide the perception of trust in the bi-factor ESEM of interpersonal trust, mostly in terms of how the other person behaves (e.g., loyal, competent, caring, and honest), the dimensions were less frequently able to explain variance in the observed items. One interpretation of these findings could be that a general sense of trust is the key factor in determining trust-specific appraisals of a known other. In the interpersonal context, opportunities to influence another’s general sense of trust may be more readily available than in the institutional context and present an effective pathway to positively (or negatively) impact perceptions of trust. How an institution or an individual might influence a person’s general sense of trust is considered below.

### Epistemic trust and the general factor

Much of the research concerned with the measurement or factor structure of trust has evolved within the realm of institutional (or organisational) trust (McEvily & Tortoriello, 2011). Considering the relevance of trust for not only our institutional interactions, but also our interpersonal ones, we saw opportunity for the theory of epistemic trust to provide a bi-directional exchange of knowledge, as this theory has been cultivated within the interpersonal context but may also have relevance in the institutional context.

Our bi-factor model results supported the notion of a general factor for both the interpersonal and institutional contexts. We assert that this general factor is congruent with an explanation of epistemic trust. To trust epistemically, and consider information at a deeper level, our minds appraise incoming information for personal relevance and generalisable significance. In part, this appraisal process considers whether the person (or institution) seeking to impart social information has first attempted to understand our *personal narrative*, or imagined *sense of self*. Where this is perceived, our *epistemic vigilance*, or natural mistrust of information that may not be personally relevant or of generalisable significance, reduces, permitting our mind to take on new data and perspectives [2].

In practice, this might manifest as a person’s capacity for epistemic trust explaining how they would *tend* to respond to a survey item about how much they trust an institution or person, whilst acknowledging that epistemic trust is itself not sufficient to fully account for variance in a person’s response, and that the specific factors also play a role. In the institutional context, our level of epistemic trust would pertain to the degree to which we believe that the institution is genuinely interested in understanding our personal narrative [2]. In interpersonal relationships, our level of epistemic trust would pertain to the degree to which we believe that the other person is genuinely interested in understanding our personal narrative. In either context, this literature would suggest that one way to influence a person’s general sense of trust would be to actively work to understand their personal narrative prior to sharing any information.

For researchers, the utility of a general factor may include the ability to derive factor scores that could be used in analyses (e.g., regression-based analyses) to help better approximate how a general trust factor may be associated with key outcomes of interest. This might include potential factors that could predict general trust, such as the identification of individual differences (e.g., cultural values, personality traits, attachment styles, demographic variables). It may also allow for the identification of outcomes that are predicted by this general factor, such as a person’s willingness to act upon communicated knowledge, or to deem the information shared with them as personally relevant. The approach of using a general factor derived from multiple indicators of trust may help to overcome potential shortcomings when using only a single indicator (e.g., a perception of confidence), and in doing so, potentially improve measurement precision.

Before the interpretation of these findings can be more robustly supported, future studies will be required to better understand the mechanisms concerning how epistemic trust may influence people’s trust-related perceptions, and how this may differ across institutional and interpersonal contexts and could enlist specific measures of epistemic trust to assess how it may predict the general factor, as well as variance across trust-related dimensions.

### A brief note on procedural justice

We also identified an unexpected pattern of item loadings in the bi-factor model for interpersonal trust that we believe may also be of interest to researchers and practitioners who promote process development through the lens of *procedural justice* [40–42]. This pattern stood out because of the expertise as legal educators of the authorship, who have worked extensively with this framework [43,44]. In summary, procedural justice is a socio-psycho-legal construct that posits perceptions related to the *fairness* and *legitimacy* of, and *satisfaction* with, a process that leads to decisions about one’s life are underpinned by five conceptual pillars, namely *voice*, *consideration of views*, *trust*, *lack of bias*, and *status recognition* [44].

As visually presented in the rightmost diagram in Fig 5, the three specific factors in the bi-factor ESEM for interpersonal trust that did not explain any significant variance in the items originally ascribed to them, or any of the other items in the survey, once a general trust factor was considered, were *perceived legitimacy*, *perceived fairness,* and *perceived bias*. Each of these conceptually aligns with an element of procedural justice, namely the process pillar of lack of bias, and the process outcomes of legitimacy and fairness. Thus, our results suggest that, in an interpersonal decision-making process, where epistemic trust is present, the process may simultaneously be perceived as legitimate, fair, and free from bias. From a reverse perspective, where epistemic trust is absent, provision of a process that is perceived as legitimate, fair, and free from bias may be difficult to achieve. While the preliminary nature of these findings must be emphasised, they do provide a rationale and theoretical foundation for further interrogation.

### Considerations for trust scholars

The results from this study invite those invested in the measurement of trust – particularly using survey-based self-report methods – to consider *which* items comprise the most suitable measure of trust. Our results suggested that [1] rather than the considerable bi-variate correlations between previously established trust dimensions being interpreted as a high degree of shared variance between these variables, this variance might instead be attributable to a general factor; [2] previously described dimensions of trust may not be as conceptually distinct as thought, as our ESEMs indicated that items often did not clearly load onto their purported corresponding trust dimension. Thus, consideration must be given as to whether the items measuring trust-related constructs (e.g., *loyalty, competence*) are statistically distinguishable, and not only distinguishable via face validity; and [3] variance in each of the 30 and 44-items for the two trust contexts, respectively, could be attributed to a general factor, which may more robustly link these items together when contrasted with previously described dimensions.

Researchers may also not always have capacity to administer lengthy surveys to a population. Thus, an important question may be to identify the fewest number of items that could produce reliable and valid assessments of trust. The development of a brief and well-validated survey instrument requires a different research methodology, such as the inclusion of multiple samples and outcome variables that could be used to assess predictive validity. However, in general, multi-item assessments tend to produce more reliable estimates that single-item (or very brief) measures, though the question of *how many items is enough to measure trust?* will also vary across contexts and research purposes. Our study does not provide a resolution to this question, though evidence for a general factor, and the subsequent capacity to derive factor scores may help to guide the development of briefer instruments – such as through the identification of items with large proportions variance explained by the general factor.

### Study limitations and further direction for future research

Some limitations to our study should be noted. In the absence of a measure that has been validated to provide concurrent assessment of a person’s interpersonal trust and institutional trust, we adapted a previously constructed institutional trust scale with application to an interpersonal setting (i.e., someone known personally to the participant). Whilst helping to address a pragmatic gap for the purposes of the current study, we also acknowledge that a separate study designing, piloting, and validating measures designed to capture trust in different targets, would be warranted to better resolve any ambiguities regarding whether items function equivalently across contexts. This concept represents an important direction for future research and highlights the potential utility of examining the invariance of different trust measures across other key defining characteristics (e.g., cultural background or gender) where experiences of trust could vary. Our design does permit the comparison of model fit *across* contexts (i.e., measurement invariance), but does highlight this a potential key area for further research development about how measures of trust operate across contexts.

It should also be noted that the specific dimensions of trust measured in the current study, whilst derived from past research [17], may not constitute a complete account of all possible indicators of trust. This consideration, however, is peripheral to our current study, which was concerned with the associations between the array of previously nominated dimensions.

Finally, regarding the study sample, we recruited a convenience sample of members from the public. Further research with other members from this population, or from specific target groups, is warranted to increase the robustness of available evidence.

## Conclusions

Our findings support earlier clarifications of the relationships between dimensions of trust in the institutional trust context. Based on model fit statistics alone, a many-factor first-order model of institutional and interpersonal trust would have been deemed the best fitting model. However, the conceptual advantages offered by the introduction of a general factor, coupled with good fit statistics for both higher-order and bi-factor models, provided evidence to support the notion that the association between items used to measure trust-related dimensions may be subject to the influence of a general factor. Statistically, it was not clear whether a higher-order model or a bi-factor model was the preferred model, though there was some preliminary evidence to prefer the bi-factor approach. Further, our exploratory use of ESEM revealed that the items and factors modelled as conceptually distinct in CFA approaches may be less distinct than initially thought. Future studies may wish to further explore and refine measures of specific trust dimensions. In the meantime, this study provided evidence indicating that significant variance in all items measuring both institutional and interpersonal trust could be explained by a general factor. This effect appeared more persuasive in interpersonal contexts than institutional contexts, where specific trust-related factors were also important. We proposed the theory of epistemic trust as a candidate for interpreting the general trust factor in both contexts. Finally, the calculation of general factor trust scores (e.g., measures of epistemic trust) may provide further insight regarding the relevance of a general trust concept across both institutional and interpersonal settings.
